# Manual of Nursing

**Published:** 1911-03

**Authors:** 


**Manual of Nursing.** By **Margaret Frances Donahoe,** formerly Superintendent of Nurses and Principal of Training School, Philadelphia General Hospital. 12 mo, pp. 489. Illustrated. New York and London: D. Appleton & Co. 1910. (Cloth, $2.00.)

As the result of experience gained while superintendent of a training school for nurses, the author of this manual has given us a well-planned and authoritative book. Directness of information, simplicity of style and a careful selection of material are characteristics of the work. The chapters on "Wounds," "Bandaging," and "Emergencies," are worthy of a place in any book on minor surgery.

Since the publication of her book Miss Donahoe married Mr. James P. Nichol, a prominent and wealthy citizen of Philadelphia. Congratulations are in order and it is fair to presume that there will be no revision by the author of her excellent manual.

